# Surgical Clinic of Prof. Gunn

**Published:** 1871

**Authors:** 


Article III.—*Surgical Clinic of Prof. Gunn.* Reported by Dr. O'Brien.

(Inserted in place of our regular notes, which were lost by the fire.)

*Saturday, Sept.* 30, 1871. Case 1. Web fingers; operation. Prof. Gunn establishes a foramen at root of web, allows it to heal through from dorsal surface to palm, getting continuity of integument there, and so preventing wound from healing up from bottom when rest of web is subsequently divided; if web were simply divided and sides separated, it would fill up from bottom and with difficulty be restored.

Case 2. Miss B. Strabismus; right eye turning in. "Ascertain cause, make careful diagnosis in these cases as between traumatic and other causes." History of this case: she says she had convulsions when young; trouble not always in same eye. "We have here, then, absence of mechanical cause, so it is not cicatrix. Remember the independent nervous supply of the external rectus, it may be paralyzed; if so, let patient try to turn the eye outward and she will be conscious of no attempt of the muscle to move; experimenting with this patient she feels the muscular effort, therefore the external rectus is not paralyzed. Having excluded these conditions, our operation is clearly indicated; we now select the eye to be operated on, and shall be content with improvement; awaiting an operation upon the other eye to complete the cure." Selecting the left eye, Prof. G. raised the conjunctiva, opened it, drew forward the internal rectus and divided it; result of the operation, perfect parallelism of the two eyes, and, so far, no second operation required.

Case 3. Eda R., aet. twelve weeks. Talipes varus of right foot. "When should we operate? Just at the time when the patient is inclined to bear his weight on feet: they will be larger, and patient will bear it better." Prof. G. usually operates when patient is six to nine months old.

Case 4. Catherine M., aet. 6. "On moving right femur we move the whole pelvis, the spinal column seeming to be the centre of motion;" (patient walked before the class, showing the characteristic swing of the whole pelvis along with the limb); "case therefore, one of hip joint disease." History: cause, a fall; been treated for pain in the knee. Prof. G., tracing the insidious onset of the disease under consideration, reminded the class of the anatomical reason why pain is first referred to the knee, when it should only furnish an index to point out the real difficulty. Enumerating the means of diagnosis, he referred to the involuntary fixing of thigh and its characteristic effect upon the gait in first stage; to the lengthening and eversion in the second; and the inversion and shortening, simulating dislocation, in the third; together, in the latter stage, with usual suppuration and abscess.

Treatment: (pain is a prominent symptom, there is no refreshing sleep.) In first stage, simply fix the joint, using dextrine or starch bandage; give *rest*, the first demand of nature in inflammation; use constitutional treatment in accordance with the state of the child; cod oil, animal food, fresh air, etc. In second stage, straighten leg, using chloroform if necessary; break up adhesions, extend leg with pully, etc., and if you have a good airy room, this treatment may be the best you can give; if you prefer to send patient into open air, put on proper splints, but splints, generally, become painful. Many cases do badly under any treatment; then resect; it promises more than anything else. Our case here requires dextrine splint; it will be applied, and patient put in charge of dispensary staff.

Case 5. Referred to Medical Department.

Case 6. P. K., aet. 35. Abscess at side of rectum; probe does not get into rectum, but the abscess probably entered into it. (Prof. G. described here fistulæ, complete and incomplete); passing bistoury on director to the point reached by probe, he cut out, not into the rectum, because fistula could not yet be traced into it, but at next examination the farthest point of this exploration will be taken as a new departure and fistula traced to its termination; will then, probably, cut into rectum, and plug.

Case 7. Eddie C., aet. 7; traumatic swelling, anterior femoral region; a good opportunity for class to make a diagnosis very frequently called for in practice. Is there constipation? No. Then it is not hernia; it feels and looks like abscess. Prof. G. opens it, giving exit to a large quantity of pus. "If abscess walls are thick, look out for femoral artery; if you fear aneurism, use exploring needle."

Case 8. Mary H., aet. 17; hare-lip. Imperfect operation at age of three months; "another instance where operation might have been postponed for some months;" lower parts of lip still joined, but central portion forming a large unsightly hiatus; left ala of nose drawn away on the cheek. "If, in operating for hare-lip we make straight incisions, there will be left a receding angle at free end of cicatrix, but if we make properly curved incisions, we can leave redundant tissue there." Prof. G. operated; cutting out cicatriced edges, but not dividing the slight point of union below; introduced silk sutures, bringing parts together, ala of nose previously spread out on cheek, coming up to its place handsomely.

Case 9. John M., aet. 6; enlarged humerus, and fistula; had pieces of necrosed bone removed at former clinic; has necrosed bone now, which will be removed at future operation.

[Reporter did not attend subsequent clinic, not anticipating that these scraps might be useful.]

